# CAA and ARIA: Emerging Concepts and Opportunities for Translation

**DOI:** 10.1002/alz.085576

**Published:** 2025-01-03

**Authors:** Steven M. Greenberg

**Affiliations:** ^1^ Massachusetts General Hospital, Boston, MA USA

## Abstract

Two broad classes of mechanisms have emerged for understanding the Amyloid‐Related Imaging Abnormalities (ARIA) associated with anti‐beta‐amyloid immunotherapy. One set of mechanisms proposes that ARIA is driven by large‐scale transfer of antibody‐bound amyloid from brain parenchyma to the perivascular and vascular compartments. This class of mechanisms is indirectly supported by neuropathological evidence that immunotherapy substantially clears plaque amyloid while increasing vessel‐associated amyloid, but has been difficult to directly demonstrate.

The second set of mechanisms proposes that ARIA is substantially driven by direct antibody binding to vascular amyloid deposits (cerebral amyloid angiopathy or CAA). The role of CAA in generating ARIA is supported by observations on propensity of transgenic mouse models with vascular amyloid to develop ARIA. Another line of supporting evidence comes from the striking neuroimaging similarities between ARIA and the spontaneous occurring syndrome of CAA‐related inflammation, a clinical entity that appears to be triggered by generation of anti‐amyloid autoantibodies. This class of mechanism predicts that CAA present prior to initiating immunotherapy should increase ARIA risk, a hypothesis supported by recent identification of cerebral microbleeds and cortical superficial siderosis on baseline MRI as independent risk factors for subsequent ARIA.

Identifying causative pathways to ARIA—the role of preexisting CAA in particular—should provide a blueprint towards future trials aimed at preventing or mitigating ARIA’s clinical impact.

